# Immunoendocrine response to individual or combined exposure of polystyrene nanoplastics and elevated salinity on gilthead seabream

**DOI:** 10.3389/fendo.2026.1759877

**Published:** 2026-03-18

**Authors:** Nuria Ruiz, Manuel Blonç, Asta Tvarijonaviciute, Mariana Teles, Josep Pastor, Lluís Tort

**Affiliations:** 1 Department of Cell Biology, Physiology and Immunology, Universitat Autónoma de Barcelona, Bellaterra, Spain; 2 Interdisciplinary Laboratory of Clinical Analysis Interlab, Universidad de Murcia, Murcia, Spain; 3 Department of Animal Medicine and Surgery, Universitat Autònoma de Barcelona, Bellaterra, Spain

**Keywords:** combined stressors, emergent pollutant, immune genes, PSNPs, *Sparus aurata*, stress response

## Abstract

The gilthead seabream (*Sparus aurata*) is one of the most important fish species in marine aquaculture, directly affected by different aspects of the “triple planetary crisis” (environmental contamination, loss of biodiversity, and climate change). This study aimed to elucidate the impact of two major components of this crisis, namely, elevated water salinity—as a direct consequence of climate change—and nanoplastics (NPs) pollution. The mucosal and systemic responses of juveniles (*S. aurata*) to exposure to high salinity and polystyrene (PS) NPs (PSNPs) both alone and in combination were assessed by analyzing the expression of relevant endocrine and immune genes in mucosal barriers (skin, gills, and intestine), as well as hematological and biochemical parameters in plasma. The results indicated tissue-specific responses to the experimental conditions, with intestine being the most responsive organ. Gills and skin were more heavily affected by exposure to salinity and PSNPs alone, respectively, and, in both cases, the combination of both challenges had a major impact compared with individual stressors. Similarly, significant hematological [white blood cell (WBC) and platelet (PLT) count] and biochemical [adenosine deaminase (ADA)] alterations occurred upon exposure to both stressors combined. Overall, the challenges induced the activation of the stress response of exposed fish, and elicited endocrine and antioxidant responses, particularly when exposed to the combination of high salinity and PSNPs. Altogether, this study highlights the role of mucosal surfaces when dealing with environmental and chemical stressors, and the importance of conducting co-exposure experiments to obtain a deeper, more realistic understanding of what aquatic organisms experience when challenged with several stressors conjointly.

## 1 Introduction

The gilthead seabream (*Sparus aurata*), a protandrous marine species, is one of the most economically important fish in the Mediterranean area, being widely reared in aquaculture facilities throughout the region (1). Given its high adaptability, its euryhaline (reportedly able to survive in salinities ranging from 2 to 60 psu; 2) and eurythermal nature (3), and its tolerance to high stocking densities, the production of this species by the aquaculture industry has triplicated since the beginning of the century, making it the third most widely produced marine fish (4). Therefore, the production of *S. aurata* plays a major role in safeguarding regional and global food supply and security.

Marine environments continuously face a variety of challenges arising from anthropogenic activities, with the most relevant (i.e., biodiversity loss, climate change and environmental contamination) being dubbed the “triple planetary crisis” by the United Nations. Evaluating the impact of these threats on natural ecosystems is of utmost importance to understand the response of both wild and cultured organisms, to predict their consequences on the aquaculture industry and food security, and to develop and implement prevention and mitigation strategies. One of the direct consequences of climate change on coastal regions is the change in salinity levels, with regional variations in observed trends and expected fluctuation intensities (5). Moreover, one of the primary sources of environmental contamination is plastic pollution (6). Plastic particles smaller than 1,000 nm (7), or nanoplastics, are widely recognized as ubiquitous in aquatic systems, and are believed to represent the majority of plastic contamination in the ocean (8). In some cases, the environmental concentrations of nanoplastics exceed 130 µg/L, with polyethylene (PE) and polystyrene (PS) being some of the most commonly detected polymers (9–11).

Previous research has investigated the adaptation potential of *S. aurata* to salinities as low as 0.3 psu (2) and as high as 55 psu (3, 12, 13). The impact of either elevated salinity or nanoplastics contamination on fish has already been widely studied on fish (including on *S. aurata*) (see reviews by 14, 15). On the one hand, *S. aurata* exposed to strong fluctuations in salinity have been reported to display slight changes in the concentration of circulating metabolites, as well as in the osmotic balance and expression of relevant genes, with the greatest response detected in mucosal surfaces, such as gills and intestine, which are in direct contact with the external environment (3, 12, 13, 16). Previous research exposing this species to 55 psu water suggests that, even though *S. aurata* exhibits altered immune function under hypersaline conditions, it is able to acclimatize, resulting in little to no effect on individual performance (12). Moreover, gilthead seabream challenged with hyperosmotic stress reportedly display significant increases in plasmatic metabolites (e.g., cortisol), as well as overexpression of relevant genes (e.g., *mr* and *hsp70*) shortly after exposure (16), and this species is able to recover basal values over time (3).

On the other hand, previous research has demonstrated that waterborne virgin PS nanoplastics (PSNPs) lead to slight changes in hematological parameters, as well as alterations in the intestine microbiome and overall metabolome in this species, causing a downregulation of genes related to antioxidant defenses and lipid metabolism (17, 18).

However, in natural environments, wild animals unlikely experience single, isolated stressors, being rather prone to encountering multiple challenges at once. Co-occurring stressors might have additive or synergistic effects, significantly worsening their impact on natural ecosystems. Thus, investigating the combined effects of high salinity and PSNPs is of utmost importance to elucidate the repercussions of these two common environmental stressors (both being directly linked to the triple planetary crisis), allowing for the obtainment of results of greater representativeness of real-life scenarios.

In fish, mucosal surfaces represent the first line of defense against external stressors such as pathogens, fluctuations in environmental parameters, and environmental contaminants, playing a major role in the ability of individuals to cope with physical and chemical threats (19, 20). To the best of the authors’ knowledge, a single study to date has focused on the combined impact of these stressors on fish, using the freshwater species *Oreochromis niloticus* as a model (21). Presumably, results obtained from challenges to salinity fluctuations and environmental contaminants will vary greatly between marine and freshwater species, considering their intrinsic differences in osmoregulatory mechanisms. Thus, the cited work does not necessarily serve as a basis for the present study. Instead, salinity levels and PSNPs concentrations were selected by investigating the available literature on research exposing *S. aurata* to these stressors. Therefore, the present study is the first to investigate both the isolated and combined effects of prolonged exposures (i.e., 28 days) to elevated salinity (i.e., 55 psu) and waterborne PSNPs (100 µg/L). To this end, variations in hematological parameters, plasma biochemistry [i.e., glucose, cholesterol, triglycerides, and adenosine deaminase (ADA)], and the expression of endocrine genes involved in the primary stress response (i.e., *mr, gr1*, and *hsp70*), antioxidant defenses (i.e., *cat* and *sod*), and immune responses (i.e., *tnfα, il10*, and *il1β*) of different mucosal surfaces (i.e., skin, gills, and intestine) in response to these stressors, both alone and in combination, were assessed. In this regard, it is hypothesized that although the gilthead seabream is expected to efficiently cope with increased salinity levels over time, the presence of environmentally relevant PSNPs concentrations might hinder this ability, triggering a myriad of responses in *S. aurata*, including stress, antioxidant, and immune mechanisms.

## 2 Materials and methodology

### 2.1 Fish and husbandry, bioassay, and sampling

**Figure 1:**
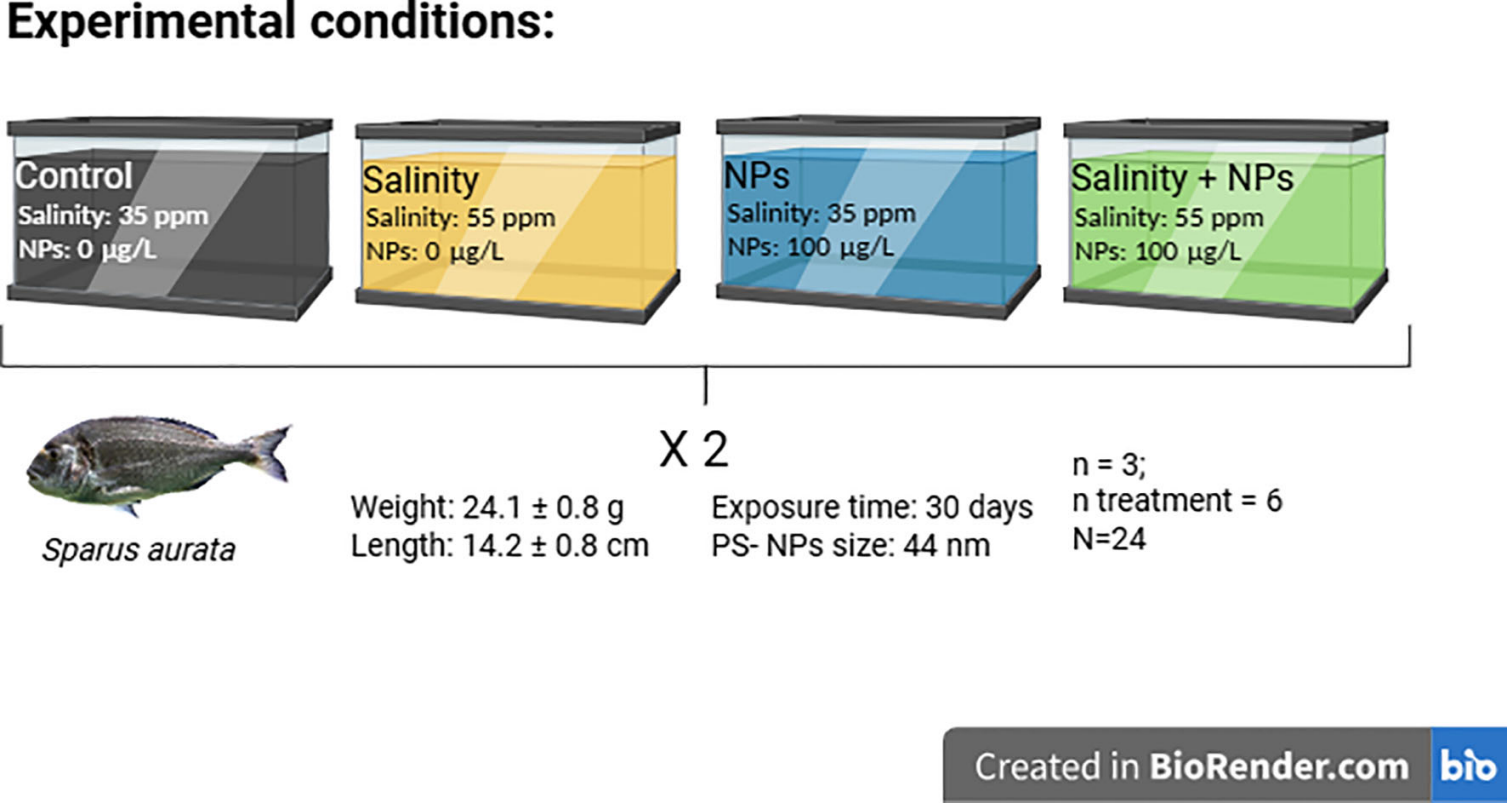
Graphical representation of the experimental design.

A total of 24 gilthead seabream (*S. aurata*) with a mean weight of 24.1 ± 0.8 g and a mean length of 14.2 ± 0.8 cm were obtained from a nearby aquaculture facility (Avramar, Burriana, Spain) and acclimated to a recirculating aquaculture system (RAS) in the facilities of the Autonomous University of Barcelona (AQUAB) for 1 month. Individuals were randomly placed in the experimental tanks subjected to four different conditions: “Control” (0 µg/L PSNPs, 35 psu), “PSNPs” (100 µg/L PSNPs, 35 psu), “Salinity” (0 µg/L PSNPs, 55 psu) and “Salinity + PSNPs” (100 µg/L PSNPs, 55 psu) (**Figure 1**). For the experiment, 40 nm of PSNPs were obtained from Bangs Laboratories Inc. (Fishers, IN, USA).

Throughout the experimental period, fish were fed until satiation at the same hour every day with a commercial diet (Skretting, Spain). The physicochemical parameters of the water were kept at optimal levels for the studied species. Regarding the procedure of PSNPs exposure, 75% of water was replaced every 48 h, after which PSNPs were added again in order to ensure the exposure concentration remained constant throughout the challenge, minimizing the impact of particle aggregation and internalization.

At the end of the experimental period, all animals were euthanized by overdose of tricaine methanesulfonate [MS222; 300 mg/L buffered with 600 mg/L (NaHCO_3_)] through immersion in a bath. Each fish was subsequently measured and weighed, and blood was extracted. Gills, skin, and intestine were then extracted from all fish (*n* = 6, *N* = 24) and snap-frozen with liquid nitrogen and stored at −80°C until analysis.

The entirety of the experiment was carried out following the 3 Rs of Animal Experimentation (Replacement, Reduction, and Refinement), under Spanish legislation (law 32/2007 and RD53/2013), and in agreement with the International Guiding Principles for Biomedical Research Involving Animals (EU 2010/63).

### 2.2 Characterization of nanoplastics

PSNPs of 40 nm (Bangs Laboratories Inc., Fishers, IN, USA) were characterized in different media, (i.e., Ultra-pure Milli-Q water and artificial saltwater at 35 and 55 psu) using dynamic light scattering (DLS; Zetasizer Pro, Malvern Instruments) to obtain particle size and polydispersity index (PDI). To this end, serial dilutions of the stock solution (100 mg/mL) were performed to obtain the experimental concentration (100 µg/L) and measurements were run five times at 25°C. PDI was automatically calculated by the software as the ratio of the weight-average molecular weight to the number-average molecular weight, and values over 0.2 were considered an indicator of particle aggregation.

### 2.3 Hematological analyses

Blood collection was performed using a heparinized syringe through caudal puncture. Blood (500 µL) was placed in Eppendorf tubes with heparin (1:40) for the hematological analysis. After this analysis, the blood was centrifuged at 2,000 *g* for 10 min at 4°C and plasma was kept at −20°C until biochemical analysis.

Hematological analyses were analyzed within 2–12 h after blood sampling by inserting 90 µL of heparanized blood into the automated flow cytometer blood cell analyzer Sysmex XN-1000V (Sysmex Corporation, Kobe, Japan). This analyzer was validated for fish and adapted from an earlier version used for avian blood analysis software. Leukocyte and erythrocyte counts, hemoglobin, hematocrit, mean corpuscular volume, mean corpuscular hemoglobin, and mean corpuscular hemoglobin concentration were determined.

### 2.4 Biochemical analyses

Cholesterol (COL), triglycerides (TG), and glucose (GLC) were determined in the plasma of fish using commercially available kits (Olympus Systems Reagents; Olympus Life and Material Science Europe GmbH, Hamburg, Germany) following the manufacturer’s guidelines. Intra- and inter-variation coefficient (CV) were below 10% in all cases. ADA activity was determined using commercially available kits (Adenosine Deaminase assay kit, Diazyme Laboratories, Poway, CA, USA) following the manufacturer’s instructions. All parameters were measured with an automatic analyzer (Olympus Diagnostica, GmbH).

### 2.5 RNA extraction and cDNA synthesis

RNA extraction was performed using the Maxwell^®^RSC simplyRNA Tissue Kit following the manufacturer’s instructions with some modifications. In brief, 15 mg of tissue of each sample was added to 400 µL of homogenization solution and homogenized using a tissue lyser (Restch MM-400) with two ceramic balls for 4 min at 25 Hz and immediately stored at −80°C for at least 24 h. After thawing the samples, 400 µL of lysis buffer and 50 µL of Proteinase K from *Tritirachium album* (20 mg/L, Sigma-Aldrich, P8044) were added. The homogenates were subsequently incubated for 10 min at room temperature and the cartridges were set into the RNA extraction equipment (Maxwell^®^RSC). Once this process was finished, the assessment of the quantity and quality (*A*_260_/*A*_280_ ratio) of the RNA was performed using a Nanodrop 2000 Spectrophotometer (Thermo Fisher Scientific Inc., USA). The cDNA of each sample was synthesized from 1 µg of RNA using the iScript™cDNA Synthesis Kit (Bio-Rad, USA) following the manufacturer’s instructions.

### 2.6 Real-time quantitative polymerase chain reaction

Gene expression was analyzed using real-time quantitative polymerase chain reaction (RT-qPCR) in a CFX Touch™Real-Time PCR Detection System (Bio-Rad, USA), according to MIQE guidelines (22). The genes analyzed in the gills, intestine, and skin were related to stress, immune, and oxidative stress responses. Regarding the stress, glucocorticoid receptor 1 (*gr1*), mineralocorticoid receptor (*mr*), and heat shock protein 70 (*hsp70*) were selected. For the immune and inflammatory responses, interleukin-1-*β*, (*il1β*), interleukin-10 (*il10*), and tumoral necrosis factor (*tnfα*) were chosen. In relation to the antioxidant for oxidative stress, catalase (*cat*), superoxide dismutase (*sod*) and peroxisome proliferator-activated receptor alpha (*pparα*) were the genes analyzed. The genes *β* actin (*β-actin*), elongation factor 1 *α* (*elf1α*), and glyceraldehyde-3-phosphate dehydrogenase (*gadph*) were employed as housekeeping genes. All pairs of primers had been previously validated in *S. aurata* tissues (23–26), and their sequence and GenBank accession number are specified in **Table 1**. Primer efficiency displayed tissue-specific values, and these are specified in **Table 2**. Prior to the analyses, a dilution curve with a pool of samples was run to determine the appropriate cDNA dilution for each gene, as well as to confirm the absence of primer dimers and the specificity of the reaction by a single peak in the melting curve for each primer set. All the analyses were performed in triplicated wells using 384-well plates with a final volume of 5 µL [2.5 µL iTAq™Universal SYBR^®^ Green Supermix (Bio-Rad, USA), 250 µM of forward and reverse primers, 250 µL of RNA-free water, and 2 µL of diluted cDNA for each sample]. The qPCR program had an initial desaturation step of 3 min at 95°C, followed by 40 cycles at 95°C for 10 s and 60°C for 30 s. The *GNorm* Software was used to calculate the best combination of two of the three reference genes tested. For the gills and intestine, the genes selected were *βactin* and *gadph*, whereas *elf1α* and *βactin* were the most suitable in skin samples. Then, the expression level of each target gene was calculated following the Pfaffl method (27).

**Table 1:** Primer sequences used in the experiment. Forward (FW) and reverse (RV) sequence (3′-5′), accession number, and the reference where the sequence were obtained are shown.

Gene	Sequence	Accession number	Reference
*gadph*	FW: TGCCCAGTACGTTGTTGAGTCCAC	DQ641630	(25)
	RV: CAGACCCTCAATGATGCCGAAGTT		
*elf1α*	FW: CCCGCCTCTGTTGCCTTCG	AF184170	(25)
	RV: CAGCAGTGTGGTTCCGTTAGC		
*βactin*	FW: TCCTGCGGAATCCATGAGA	X89920	(23)
	RV: GACGTCGCACTTCATGATGCT		
*gr1*	FW: GAAGGATGGAGAGCACGACAAAA	XM_030437675.1	(26)
	RV: GCTTCCAAGTTCATTCCGGC		
*mr*	FW: CGCCTGGCTGGAAAGCAGATG	XM030418022.1	(26)
	RV: GAGGTCAGGGGCAAAGTAGAGCAT		
*hsp70*	FW: AGGTTGGGTCTGAAAGGAAC	EU805481.1	(26)
	RV: TGAACTCTGCGATGAAGTGG		
*il*1*β*	FW: TCAGCACCGCAGAAGAAAAC	AJ277166.2	(26)
	RV: TAACACTCTCCACCCTCCAC		
*tnfα*	FW: TCGTTCAGAGTCTCCTGCAG	AJ413189.2	(24)
	RV: AAGAATTCTTAAAGTGCAAACACACCAAA		
*il10*	FW: AACATCCTGGGCTTCTATCTG	JX976621	(24)
	RV: TGTCCTCCGTCTCATCTG		
*cat*	FW: TGGTCGAGAACTTGAAGGCTGTC	JQ308823	(25)
	RV: AGGACGCAGAAATGGCAGAGG		
*sod2*	FW: CCTGACCTGACCTACGACTATGG	JQ308833	(25)
	RV: AGTGCCTCCTGATATTTCTCCTCTG		
*pparα*	FW: GCACCTGTGAGTCTTGTGAGTGA	AY590299	(25)
	RV: CTCCATCAGGTCTCCACACAGC		

**Table 2:** Efficiencies of the selected primers.

Gene	Gills (%)	Skin (%)	Intestine (%)
*gadph*	93.9	94.2	96.6
*elf1α*	96.4	96.3	90.7
*βactin*	91.8	93.2	97.1
*gr1*	94.1	100.5	103.5
*mr*	94.7	94	93.1
*hsp70*	95	92.7	99
*il1β*	103.2	108.9	90
*tnfα*	96.4	92.2	92.5
*il10*	106.1	108.5	98.8
*cat*	95.8	92.5	90.4
*sod2*	90.4	101.9	90.6
*pparα*	102.2	93.9	108.2

### 2.7 Statistical analysis

All data were analyzed using the RStudio software (https://www.R-proyect.org) software. The data and residuals were fitted into the Gaussian family, and normality and homoscedasticity were tested by Shapiro–Wilk and Bartlett tests, respectively. Data that met parametric assumptions were analyzed with analysis of variance (ANOVA) tests, whereas data that did not meet the requirements were analyzed with GzLM. Appropriate pairwise comparisons were applied by Tukey’s correction if significant differences were found (*p*-value *<* 0.05). Results are shown as mean ± standard errors (SEM, *n* = 6, *N* = 24). Data were represented using the GraphPad Prism 8 Software. In tables, data are shown as the mean ± standard deviation.

## 3 Results

In this section, the results of the characterization of PSNPs and the effects on the fish are presented. Plasma results are divided into two groups since there were two different types of analysis. Because of tissue-specific responses, the results are presented separately for each organ.

### 3.1 Characterization of nanoplastics

DLS characterization of PSNPs revealed clear differences in particle size between ultrapure, fresh, and ASW, although no differences emerged between ASW and hypersaline media (**Table 3**). PSNPs in ultrapure-Milli-Q water showed less dispersion in size than those in ASW. A PDI value over 0.2, as observed in ASW, indicates that the particles in this medium aggregated, as reflected by the increase in size. Nonetheless, the characterized particles remained, in all cases, in nanoscale size.

**Table 3:** Characterization of polystyrene nanoplastics (100 µg/L) in ultrapure-Milli-Q water and artificial salt water at 35 and 50 psu.

Water	Size	Polydispersity index (PDI)
Ultra-Pure Milli-Q water	47.37 ± 1.03	0.06 ± 0.006
Artificial saltwater 35 psu	557 ± 67.07	0.98 ± 0.115
Artificial saltwater 55 psu	516 ± 90.01	0.72 ± 0.205

Measured parameters were size (nm) and polydispersity index (PDI) shown as mean ± standard deviation.

### 3.2 Hematological analyses

The results obtained from the automated hematological analyzer indicated no significant differences in any of the investigated parameters following the chronic waterborne exposure to PSNPs and salinity except in the case of the thrombocytes and white blood cell (WBC) count (**Table 4**). Thrombocytes showed a decrease in the case of PSNPs and salinity + PSNPs compared to the control. In the case of the WBC, an increase in the salinity group compared with the control was observed.

**Table 4:** Hematological parameters of *Sparus aurata* exposed to polystyrene nanoplastics and salinity, both alone and in combination for a 28-day period (Control: PSNPs = 0 µg/L, salinity = 35 psu; PSNPs: PSNPs = 100 µg/L, salinity = 35 psu; salinity: PSNPs = 0 µg/L, salinity = 55 psu; salinity + PSNPs: PSNPs = 100 µg/L, salinity = 55 psu).

Parameter	Control	Salinity	Nanoplastics	Salinity + Nanoplastics
RBC (10^3^/µL)	2.34 ± 0.005	2.68 ± 0.36	2.47 ± 0.29	2.77 ± 0.20
WBC (10^3^/µL)	55.85 ± 6.28 *^b^*	72.44 ± 3.98 *^a^*	64.01 ± 14.71 *^ab^*	71.40 ± 9.33 *^ab^*
HGB (g/dL)	4.82 ± 0.65	5.75 ± 1.08	5.55 ± 0.71	6.06 ± 0.76
HCT (%)	39.30 ± 1.20	51.83 ± 11.19	46.43 ± 6.49	53.16 ± 10.02
Heterophils (%)	7.96 ± 1.53	8.08 ± 1.33	8.70 ± 1.42	7.67 ± 0.59
MCHC (g/dL)	11.34 ± 1.48	11.17 ± 1.08	12.00 ± 0.57	11.52 ± 1.13
PLT (10^3^/µL)	4.40 ± 1.81 *^a^*	3.00 ± 1.26 *^ab^*	1.50 ± 1.05 *^bc^*	1.00 ± 0.00 *^c^*
RDW (fL)	14.28 ± 1.10	14.68 ± 2.33	12.47 ± 1.10	15.74 ± 3.09
MCV (fL)	145.3 ± 4.45	168.4 ± 14.54	163.6 ± 13.03	168.4 ± 30.56
MCH (pg)	17.46 ± 2.11	18.77 ± 1.57	19.60 ± 1.48	19.18 ± 2.24

Expressed as group mean ± standard deviation. RBC, erythrocyte count; WBC, leukocyte count; HGB, hemoglobin; HCT, hematocrit; Heterophils %; MCHC, mean corpuscular hemoglobin concentration; PLT, thrombocyte count; RDW, red cell distribution width MCV, mean corpuscular volume; MCH, mean corpuscular hemoglobin. Letters mean significant differences between groups. Different letters indicate significant differences (p<0,05).

### 3.3 Biochemical analyses

Regarding biochemistry, no differences were found in glucose, triglycerides, and cholesterol (**Table 5**). In the case of the ADA, the combination of both stressors produced a significant increase compared to the PSNPs group, whereas each individual stressor and the combination of both did not differ from the control.

**Table 5:** Biochemical parameters of *Sparus aurata* exposed to polystyrene nanoplastics and salinity, both alone and in combination for a 28-day period (Control: PSNPs = 0 µg/L, salinity = 35 psu; PSNPs: PSNPs = 100 µg/L, salinity = 35 psu; salinity: PSNPs = 0 µg/L, salinity = 55 psu; salinity + PSNPs: PSNPs = 100 µg/L, salinity = 55 psu).

Parameter	Control	Salinity	Nanoplastics	Salinity + Nanoplastics
GLC (mg/dL)	67.87 ± 1.626	82.70 ± 11.30	71.67 ± 9.38	86.10 ± 12.78
TG (mg/dL)	176.7 ± 20.24	232.6 ± 55.50	269.6 ± 62.12	198.8 ± 74.69
ADA (IU/L)	5.18 ± 1.49*^ab^*	4.52 ± 1.03*^ab^*	3.89 ± 1.11*^b^*	11.58 ± 8.52*^a^*
COL (mg/dL)	321.5 ± 42.58	359.8 ± 30.29	345.3 ± 49.98	38.2 ± 57.00

Expressed as group mean ± standard deviation. GLC, glucose; TG, triglycerides; ADA, adenosine deaminase; COL, cholesterol. Letters mean significant differences between groups. Different letters indicate significant differences (p<0,05).

### 3.4 Gills gene expression

**Figure 2:**
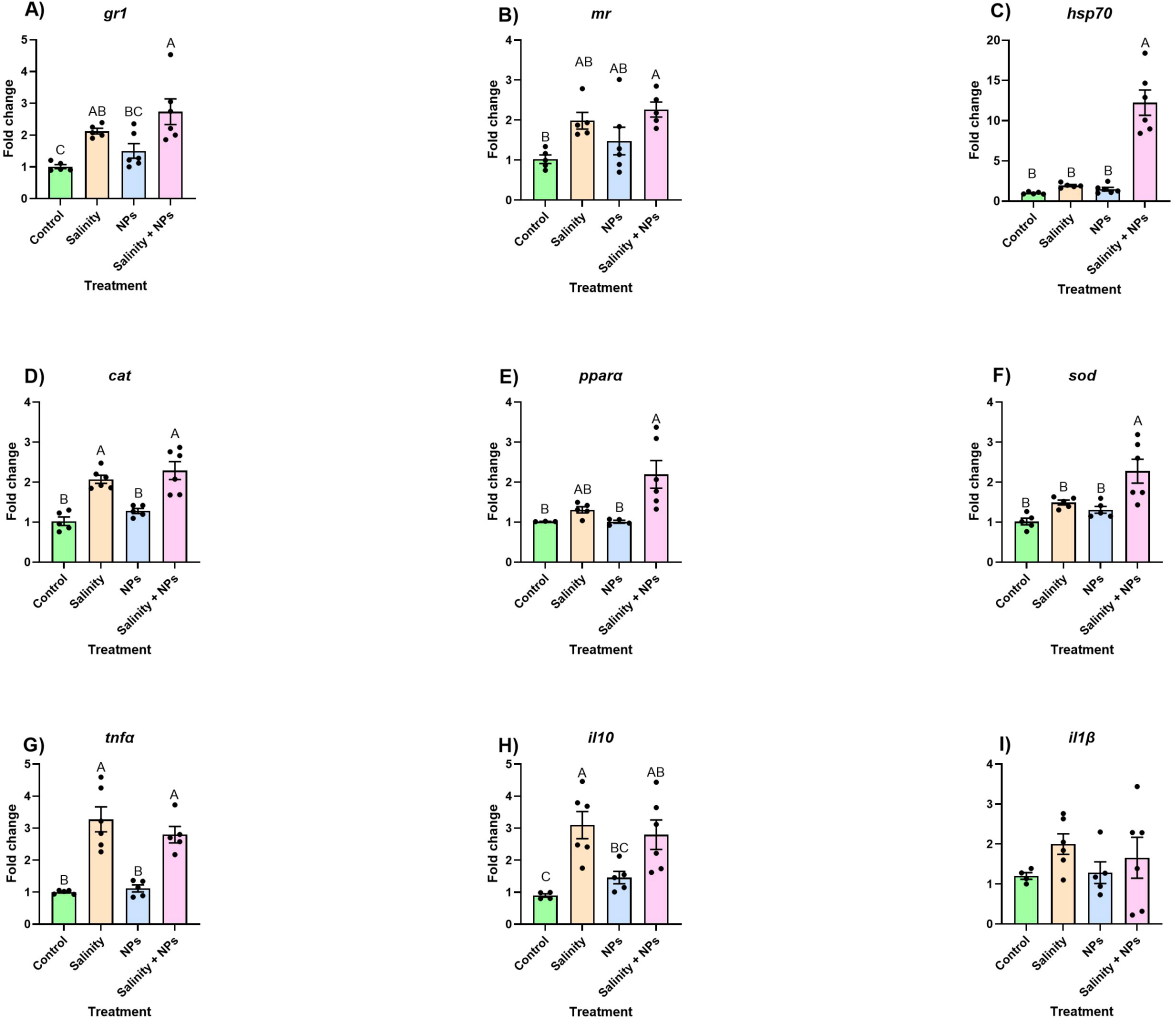
Relative expression of **(A)** glucocorticoid receptor 1 (gr1), **(B)** mineralocorticoid receptor (mr), **(C)** heat shock protein (hsp70), **(D)** catalase (cat), **(E)** peroxisome proliferator-activated receptor alpha (pparα), **(F)** superoxide dismutase (sod), **(G)** tumor necrosis factor alpha (TNFα), **(H)** interleukin 10 (IL-10), and **(I)** interleukin 1 beta (IL1β) in gills of fish exposed to 28-day waterborne exposure to salinity and nanoplastics. Data are represented as mean ± standard error (n = 6 per group). Significant differences (p < 0.05) are shown with different letters. Individual values are plotted as dots in the figure.

The expression of the analyzed genes displayed similar patterns between genes in an organ-specific manner. In the case of *gr1*, the PSNPs group did not differ from the control group, but overexpression was observed in the salinity and salinity + PSNPs compared to the control, with the group exposed to both stressors displaying the greatest fold change (**Figure 2A**). Similarly, *mr* became upregulated to some extent in all exposed groups. Although only the combination of both stressors yielded results significantly different from the control, these were not significantly different from those observed in groups exposed to individual stressors. In other words, stressors alone showed intermediate values between control and the combined stress (**Figure 2B**). Lastly, the expression of *hsp70* did not show any significant changes between groups exposed to individual stressors and the control. However, and similarly to what was described for *mr*, the combination of both stressors resulted in the upregulation of this gene with a fold change of over 3, compared to all other experimental groups (**Figure 2C**).

As for the oxidative stress response, the expression of the genes followed a similar pattern to those related to the generalized stress response, with the group exposed to combined stressors displaying the highest degrees of upregulation (**Figures 2D–F**). Regarding *cat*, only the salinity and salinity + PSNPs groups were upregulated compared to the control, but they were not significantly different from each other (**Figure 2D**), and no changes were observed between the PSNPs and the control group. In a similar manner, the expression of *pparα* in the PSNPs group did not significantly differ from that in the control. As observed with the expression levels of other genes analyzed in the present study, the group exposed to the combination of high salinity and PSNPs displayed a significant upregulation compared to the control (**Figure 2E**). Similarly, the mRNA abundance of *sod* only became significantly higher in the group exposed to the combined stressors, with no other significant differences arising (**Figure 2F**).

The response of immune-related genes somewhat differed from the patterns observed in the other gene classes, with salinity causing the highest degrees of regulation. In the case of *tnfα* salinity alone, and in combination with PSNPs, both caused a significant increase in mRNA abundance, whereas PSNPs alone did not have any effect (**Figure 2G**). Similarly, *il10* was significantly upregulated in the high-salinity group. Interestingly, the expression of this gene did not differ between the PSNPs and the control group, and the combined effect of PSNPs and high salinity appeared less severe than that observed in the group exposed solely to high salinity. Finally, the PSNPs group was a bit upregulated, but it was not different from the control (**Figure 2H**). Lastly, regarding *il1β*, no differences were found between groups (**Figure 2I**).

### 3.5 Intestine gene expression

**Figure 3:**
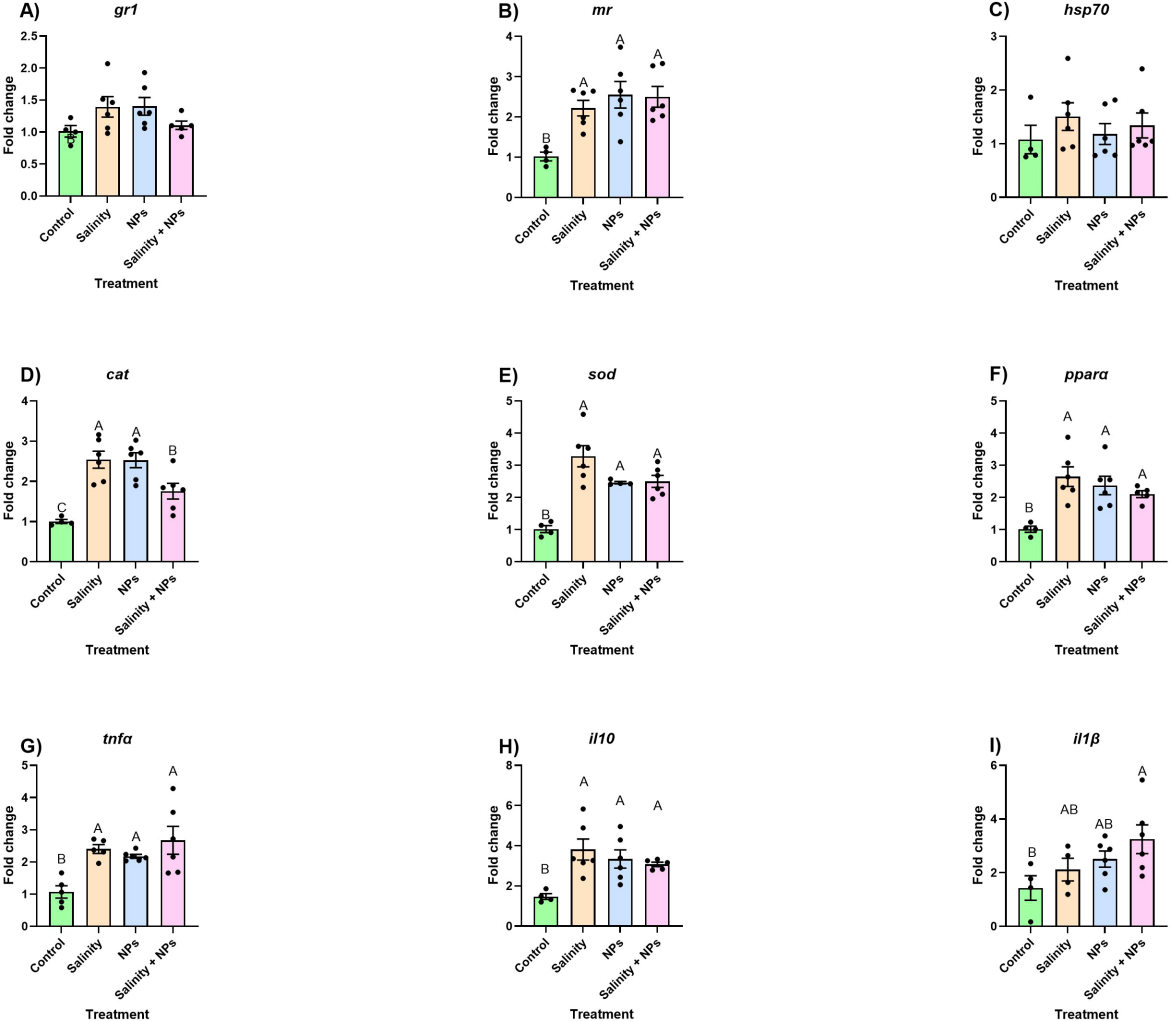
Relative expression of **(A)** glucocorticoid receptor 1 (gr1), **(B)** mineralocorticoid receptor (mr), **(C)** heat shock protein (hsp70), **(D)** catalase (cat), **(E)** peroxisome proliferator-activated receptor alpha (pparα), **(F)** superoxide dismutase (sod), **(G)** tumor necrosis factor alpha (TNFα), **(H)** interleukin 10 (IL-10), and **(I)** interleukin 1 beta (IL1β) in intestine of fish exposed to 28-day waterborne exposure to salinity and nanoplastics. Data are represented as mean ± standard error (n = 6 per group). Significant differences (p < 0.05) are shown with different letters. Individual values are plotted as dots in the figure.

In the intestine, the only gene related to the generalized stress response that became significantly altered by the experimental conditions was *mr* (**Figures 3A–C**), being significantly upregulated in all exposed groups compared to the control, but without any differences between each other (**Figure 3B**).

Regarding the oxidative stress, a generalized upregulation of the stressors alone or in combination compared with the control group was observed (**Figures 3D–F**). In the case of *sod* and *pparα*, the combination of stressors had a similar impact to individual stressors (**Figures 3E, F**). In the case of *cat*, all exposed groups induced significant increases, although individual stressors resulted in greater upregulation compared to the combined exposure (**Figure 3D**).

Finally, in the case of the immune response, *tnfα* and *il10* showed an upregulation in the stressed groups compared with the control, and no differences were observed between single or combined stressors (**Figures 3G, H**). The results of *il1β* showed that the combination of both stressors led to the upregulation of this gene, with measured values being significantly different from the control. Nevertheless, each stressor alone had a light impact on the expression of this gene, leading to its slight overexpression, resulting in intermediate values between control and the combined exposure (**Figure 3I**).

### 3.6 Skin gene expression

**Figure 4:**
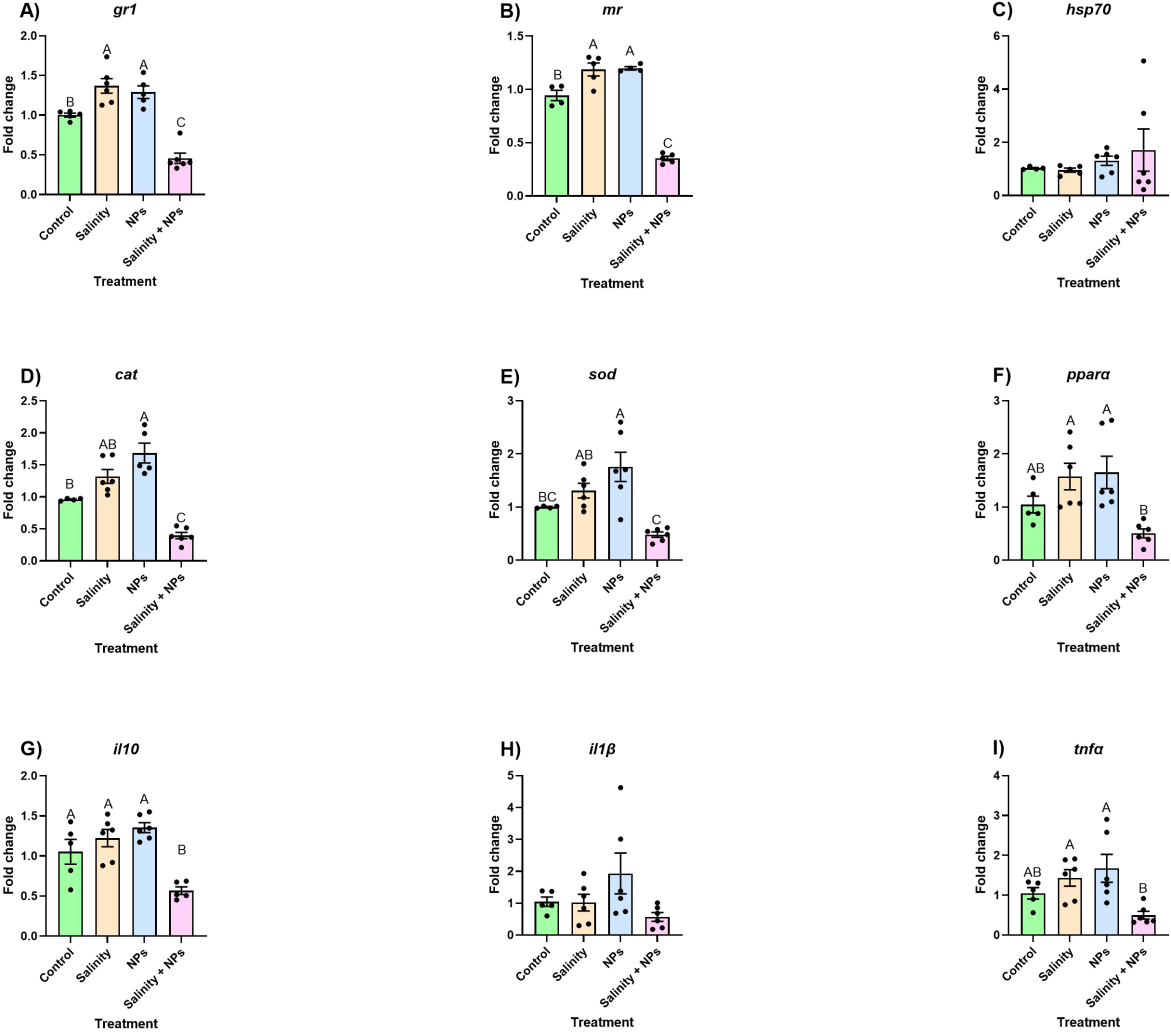
Relative expression of **(A)** glucocorticoid receptor 1 (gr1), **(B)** mineralocorticoid receptor (mr), **(C)** heat shock protein (hsp70), **(D)** catalase (cat), **(E)** peroxisome proliferator-activated receptor alpha (pparα), **(F)** superoxide dismutase (sod), **(G)** tumor necrosis factor alpha (TNFα), **(H)** interleukin 10 (IL-10), and **(I)** interleukin 1 beta (IL1β) in skin of fish exposed to 28-day waterborne exposure to salinity and nanoplastics. Data are represented as mean ± standard error (n = 6 per group). Significant differences (p < 0.05) are shown with different letters. Individual values are plotted as dots in the figure.

The results obtained from the gene expression analyses of stress-related genes in skin samples revealed that, in both *gr1* and *mr*, significant upregulations occurred when exposed to either high salinity or PSNPs alone. Nevertheless, when both stressors are combined, a significant downregulation was observed compared with all other experimental groups, including the control (**Figures 4A, B**). No differences were found in the case of *hsp70*. A similar pattern occurred when investigating the response of the oxidative stress-related gene *cat*. Indeed, the abundance of this gene was significantly reduced in fish exposed to salinity + PSNPs compared with control values, and even though exposure to salinity alone had little impact on this gene, the sole exposure to PSNPs resulted in a significant upregulation of *cat* compared with all other experimental groups (**Figure 4D**). Regarding *sod*, the combination of both stressors showed downregulation compared with each stressor alone but not with the control. Moreover, the control was not different from salinity stress despite the fact that it was being slightly upregulated, whereas PSNPs displayed the strongest degree of upregulation differing significantly from the control (**Figure 4E**). Regarding *pparα*, no changes were observed compared to the control. In this case, differences were observed between treatments, with each individual stressor resulting in greater mRNA abundances of *pparα* in comparison with both stressors together (**Figure 4F**).

Finally, the immune genes in the case of *tnfα* were downregulated in the group of salinity + PSNPs compared to the control and each stressor alone (**Figure 4G**). Regarding the *il10*, no changes were observed compared to the control. As described above for *pparα*, expression of this gene was upregulated under each single-stressor condition compared to the combined exposure (**Figure 4H**). Finally, no differences were found regarding *il1β* (**Figure 4I**).

## 4 Discussion

The expression of the investigated genes in *S. aurata* juveniles, both alone and in combination, was modulated by the experimental factors, elevated salinity levels and PSNPs, and displayed tissue-specific patterns. The response of each tissue was also dependent on the stressor, showing marked differences between fish exposed to hyperosmotic stress, PSNPs, and the combination of both. Previous research demonstrated that, although different organs might display similarities in their response to the same stressor, tissue-specific responses also arise. The same patterns have been reported in other euryhaline species exposed to changes in salinity (28–30), and PSNPs (31, 32).

### 4.1 Impact of increased salinity

Abrupt changes in salinity levels have been described to cause significant changes in the measured levels of circulating metabolites (e.g., glucose and triglycerides), with basal values being recovered approximately 3 days after exposure, indicating correct acclimation (33, 34). Thus, it is likely that changes in these parameters occurred in the fish hereby challenged during the first days of the experiment but did not persist until the sampling period.

As high salinity may disrupt ion and water balance in fish mucosae causing cellular oxidative stress and local inflammation, as shown in the gene expression results, pro-inflammatory signals may produce recruitment and proliferation of phagocytes and other leukocytes, contributing to the increased WBC count. In addition, as hyperosmotic exposure activates the hypothalamus–pituitary–interrenal (HPI) axis, elevating cortisol and catecholamines, leukocytes will mobilize from hematopoietic organs (head kidney and spleen) into the bloodstream (35).

The exception is that platelets show a decrease after salinity exposure, which can be related to transient endothelial injury activating hemostasis, recruiting and aggregating thrombocytes at damaged sites and reducing them in the bloodstream, as it has been shown in fish after contaminant exposure (36).

Although such high salinity levels are not particularly common, they can occur under environments such as shallow waters or estuaries in which seabream can thrive and be cultured, and even more frequently under heat episodes driven by climate change.

In gills, hypersaline water appeared to slightly trigger the primary stress response, and to elicit a mild antioxidant response, as evidenced by the upregulation of *gr1* and *cat*. Previous research has shown that, in euryhaline species, including *S. aurata*, exposures to both low and elevated salinity levels lead to the upregulation of glucocorticoid receptor-coding genes (16, 29). In a similar note, Martos-Sitcha et al. (37) described that hyper-osmotic stress induced the activation of the HPI axis in the gilthead seabream. Similar to what was described by Zarantoniello et al. (16), the expression of *hps70* was not altered in juvenile *S. aurata* following a salinity challenge. Likewise, exposure to a hypersaline environment altered the mRNA abundance of genes related to an immune response in the gills of the studied fish. Indeed, genes coding for both pro-inflammatory cytokines *il1β* and *tnfα* were significantly upregulated in this tissue. Moreover, the anti-inflammatory *il10* also became upregulated in this context. Anti-inflammatory mechanisms were likely delayed relative to pro-inflammatory responses, acting as a negative feedback loop (38). Nonetheless, the immune response elicited by the high salinity appears successfully suppressed, allowing for maintained homeostasis (39). However, given that measurements were taken at a single time point, this cannot be verified by the present study. Future research could benefit from investigating the temporal dynamics of gene expression in the gills of gilthead seabream exposed to high salinities.

The gastrointestinal tract has been demonstrated to play a major role in coping with osmotic stress in both marine and freshwater species challenged with fluctuations in water salinity (40, 41). Previous research investigating the impact of variations in salinity on fish intestine has mostly focused on microbiome alterations, describing species-specific responses (40, 42, 43). In the analyzed intestine samples, the significant increase in the mRNA abundance of *mr*, *cat*, and *sod* corroborates the hypothesis that exposure to 55 psu induced both stress and antioxidant responses in juvenile *S. aurata*. Moreover, the immune-related genes investigated in this tissue also became significantly upregulated, indicating a potential pro-inflammatory response to the challenge, followed by negative feedback of anti-inflammatory mechanisms, as observed in the gills. In fish, salinity changes might cause intestine dysbiosis, which has been suggested to lead to inflammatory responses in this organ (44). Furthermore, and in accordance with the present results, hyperosmotic stress has been reported to modulate the expression of intestinal genes involved in both pro- and anti-inflammatory processes, and antioxidant defenses, as evidenced by transcriptomic analysis (45). In addition, intestine samples displayed significantly greater levels of *pparα* in the high-salinity group compared to the control, hinting towards a mild disturbance in lipid metabolism pathways, agreeing with previously published reports of hyperosmotic stress altering metabolic processes (43). However, the measured levels of circulating triglycerides and cholesterol did not significantly change from one group to another.

Differences in the response to environmental pollutants between marine and freshwater species might be partly explained by the osmoregulatory mechanisms put in place. Indeed, marine fishes, being hypo-osmoregulators, constantly lose water through their skin and gills and are therefore compelled to constantly drink water. In contrast, freshwater fish are hyper-osmoregulators and continually absorb water from the surrounding environment (46, 47). These differential osmoregulatory requirements might translate into differing rates in (nano) plastic absorption and retention, particularly through the gastrointestinal tract (48).

In the skin samples, only two genes were significantly upregulated as a result of hyperosmotic stress, namely, *mr* and *gr1*, both closely involved in the primary stress response by modulating the expression of cortisol receptors (26), indicating that this organ also plays an important role in this process.

### 4.2 Impact of waterborne PSNPs

In agreement with previous studies looking into the impact of PSNPs on the hematological profiles of *S. aurata*, no changes were observed in hematological parameters following PSNPs treatment (17, 25) or in the investigated plasmatic biochemical assays (18). None of the investigated genes in gills became significantly up- or downregulated in the face of waterborne PSNPs alone. Although previous research has shown that waterborne exposure to PSNPs might induce transcriptional changes in the gills of some fish species (49, 50), these are generally observed after exposure to particle concentrations higher than environmentally relevant and, thus, several orders or magnitude greater than the concentration employed here. To the best of the authors’ knowledge, no study has investigated the impact of PSNPs on gene expression levels in the gills of *S. aurata*, and differences in published results might, therefore, also be partly explained by species-specific characteristics. Indeed, the impact of PSNPs seems highly dependent, not only on exposure concentration but also on polymer, particle size, and the species studied (15, 51).

In contrast with what was observed in the gills, the exposure to PSNPs elicited a response in the intestine similar to that to salinity, with gene expression values significantly different from the control group, but not from the high-salinity group. In this organ, the mRNA abundance of *pparα* was also significantly greater than values from the control group, potentially hinting, in a similar manner, towards a light disturbance of the lipid metabolism, in accordance with previous reports of PSNPs altering intestinal lipid profiles (52). This could be linked to a direct disruption of the expression of peroxisome-proliferator-activated receptor (PPAR)-associated genes (53) or to the indirect impact of shifts in the intestinal microbiome (54), although no evidence to verify this hypothesis has been obtained through the present work. Moreover, as observed in the skin of fish exposed to hyperosmotic stress, the skin samples of the PSNPs-exposed group exhibited a significant upregulation of both *mr* and *gr1* compared to the control group, reaffirming the role of this organ in the primary stress response (26, 55). In addition, significantly higher levels of *cat* and *sod* were observed in this organ, indicating that antioxidant processes were also triggered in the skin (56). As observed in the intestine, no statistically significant differences arose in the gene expression levels of the skin between both groups exposed to single stressors.

### 4.3 Combined impact of increased salinity and waterborne PSNPs

Perhaps more interestingly, salinity and PSNPs appear to have had, to some extent, a synergistic effect on the gills of *S. aurata* juveniles. Indeed, only this experimental group displayed significant changes in ADA. The significant increase in this parameter might reflect the activation of an immune response, given its demonstrated role in the modulation of inflammatory and homeostatic processes in fish following exposure to stressful events (57, 58).

Moreover, out of all studied genes, only five were significantly altered in this tissue when exposed to 55 psu, and none were altered by the sole exposure to PSNPs. However, eight genes were significantly upregulated by the combination of both stressors, two of which were significantly upregulated compared to individual treatments, namely, *hps70* and *sod*. Therefore, although the upregulation of certain genes observed in the co-exposed group can be solely attributed to the effect of salinity, the combined impact of salinity and PSNPs appears to have triggered a stress and antioxidant response at a greater magnitude in the gills than that observed in single exposures. In the intestine, the combination of both stressors also appeared to have a slightly different effect than either isolated stressor. Indeed, the co-exposed group displayed a higher and a lower expression of *il1β* and of *cat*, respectively, compared to all other groups. As observed in the other tissues, the collective impact of hyperosmotic stress and PSNPs caused a greater level of disturbances in the skin than either stressor on its own. In this specific case, however, the combination of both challenges had the opposite effect than that observed for single exposures. The expression of stress-related genes, genes coding for antioxidant enzymes, and genes involved in both pro- and anti-inflammatory processes were all significantly lower in this group compared to all three other experimental groups. This might indicate, to some extent, a suppression of the immune function of the skin in juvenile *S. aurata* as a result of the experimental challenge. In this group, this contrasts with the significantly higher circulating levels of ADA, known to modulate inflammatory responses by promoting the production of T cells (59), compared to that solely exposed to PSNPs. Interactive effects of PSNPs and co-contaminants on aquatic organisms have been widely studied, generally indicating that such co-exposures result in increased toxicity (60–62). For instance, the only study investigating similar stressors to those employed in the present study in fish demonstrated that high salinity levels exacerbate the impact of PSNPs in the fish, amplifying their effect on the modulation of immune and antioxidant responses (21).

It is important to note that, given the intricacy of gene roles, interactions and expression modulation, or changes in the mRNA abundance of single genes do not necessarily translate into functional changes at the whole-organism level (63). This was observed in the present study, for example, with the somewhat contradictory results obtained from expression analysis of lipid metabolism-related genes and plasmatic levels of circulating lipids, indicating that other factors than those hereby analyzed were at play. Thus, the present results should be considered as preliminary, serving as a basis for future research investigating functional alterations on *S. aurata* exposed to hypersaline media and waterborne PSNPs. Alternatively, it could also prove interesting to look at similar scenarios in hypoosmotic conditions. In either case, future research would benefit from establishing multiple sampling points throughout the exposure, thus assessing the evolution of the response of *S. aurata* to the combination of these stressors over time, to evaluate whether habituation might occur. Similarly, investigating the combined impact of PSNPs or salinity with other environmental or chemical stressors, as well as mechanical stressors linked to the aquaculture industry (e.g., overcrowding, handling, and transportation), would allow to broaden the current knowledge available on the impact of the “triple planetary crisis” on the aquaculture industry.

## 5 Conclusion

The present study provides valuable knowledge on the endocrine, metabolic, and hematological response of mucosal surfaces in a marine fish of major economic importance to two major aspects of the “triple planetary crisis”, namely, increased salinity, as a direct consequence of climate change, and PSNPs, one of the most important components of environmental contamination, both alone and in combination. Obtained data suggest that high salinity and PSNPs had, to some extent, a synergistic impact on the hematological and biochemical profiles of the exposed fish, inducing the activation of the immune system. Moreover, the results indicate marked tissue-dependent differences in the responses to these stressors, with intestine being the most responsive organ to salinity and PSNPs, both alone and in combination, but with little difference between these. On the other hand, gills and skin were more heavily affected by exposure to salinity and PSNPs alone, respectively. In both these cases, the stressors had a major impact combined than individually. The results highlight the differential importance of mucosal surfaces when a marine fish is exposed to environmental and chemical stressors. Moreover, the present study emphasizes the relevance of investigating combined exposures to different stressors to obtain more reliable results, reflecting to a greater extent what occurs in real-life scenarios. The present study can help future studies as a foundation for research investigating the mucosal response of fish to the combined effect of stressors linked to anthropic activities.

## Data Availability

The datasets presented in this study can be found in online repositories. The names of the repository/repositories and accession number(s) can be found below: https://ddd.uab.cat (Digital Document Repository).
